# An fMRI comparison of neural activity associated with recognition of familiar melodies in younger and older adults

**DOI:** 10.3389/fnins.2015.00356

**Published:** 2015-10-06

**Authors:** Ritu Sikka, Lola L. Cuddy, Ingrid S. Johnsrude, Ashley D. Vanstone

**Affiliations:** ^1^Centre for Neuroscience Studies, Queen's UniversityKingston, ON, Canada; ^2^Music Cognition Lab, Department of Psychology, Queen's UniversityKingston, ON, Canada; ^3^Cognitive Neuroscience of Communication and Hearing, Department of Psychology, Queen's UniversityKingston, ON, Canada

**Keywords:** recognition, familiarity, fMRI, music, aging, semantic memory, melodies

## Abstract

Several studies of semantic memory in non-musical domains involving recognition of items from long-term memory have shown an age-related shift from the medial temporal lobe structures to the frontal lobe. However, the effects of aging on musical semantic memory remain unexamined. We compared activation associated with recognition of familiar melodies in younger and older adults. Recognition follows successful retrieval from the musical lexicon that comprises a lifetime of learned musical phrases. We used the sparse-sampling technique in fMRI to determine the neural correlates of melody recognition by comparing activation when listening to familiar vs. unfamiliar melodies, and to identify age differences. Recognition-related cortical activation was detected in the right superior temporal, bilateral inferior and superior frontal, left middle orbitofrontal, bilateral precentral, and left supramarginal gyri. Region-of-interest analysis showed greater activation for younger adults in the left superior temporal gyrus and for older adults in the left superior frontal, left angular, and bilateral superior parietal regions. Our study provides powerful evidence for these musical memory networks due to a large sample (*N* = 40) that includes older adults. This study is the first to investigate the neural basis of melody recognition in older adults and to compare the findings to younger adults.

## Introduction

Aging affects brain mechanisms underlying many cognitive processes including those related to memory. Even when younger and older adults perform equally well on a cognitive task, older adults may display increased or decreased neural activity in specific brain regions, depending on the actual task (Grady, 2012). Aging-related changes in memory, whether for performance or for the corresponding neural activity, may vary depending on many factors, such as type of memory (e.g., semantic, episodic), memory process (e.g., encoding, retrieval), sensory modality involved in the memory, age of the individual, and possibly age of the memory itself. Although studies of musical memory are scarce compared to those with verbal or pictorial stimuli, they suggest that musical memory may be preserved in aging and even in Alzheimer's disease (Vanstone et al., 2012; Kerer et al., 2013). However, studies of brain activity associated with musical memory have largely limited their participants to younger adults. Thus, the effects of aging on neural activity related to musical memory remain unknown.

Though, not all memories can be neatly classified into any single category, a distinction between semantic and episodic memory, as proposed by Tulving (1972), is supported by behavioral and neural evidence (Mayes and Montaldi, 2001; Tulving, 2002; Winocur and Moscovitch, 2011; Yee et al., 2013). Semantic memory is knowledge about the world, including concrete objects and abstract concepts, as well as relationships between them, whereas episodic memory involves details about the temporal and spatial context of an event. Musical memory, that is, memory of the music itself, has been considered a type of semantic memory since it comprises knowledge of the musical characteristics of a tune (i.e., the way it sounds, and not any “meta” information such as composer or title). Several studies have regarded musical semantic memory as the underlying basis of familiar melody recognition (Halpern and Zatorre, 1999; Platel et al., 2003; Schulkind, 2004; Groussard et al., 2009; Hailstone et al., 2009; Omar et al., 2010; Weinstein et al., 2011; Vanstone et al., 2012).

The ability to recognize a melody in as little as 2 s, with only 3–6 notes (Bella et al., 2003) requires fast retrieval from semantic memory. A musical lexicon, akin to the concept of a language-based lexicon, is thought to be a type of music storage system that is accessed effortlessly during recognition of familiar music (Peretz, 1996). This lexicon is assumed to be a module in a postulated system of hierarchically organized, independently functioning modules involved in the processing of music in the brain (Peretz and Coltheart, 2003). “The musical lexicon is a representational system that contains all the representations of the specific musical phrases to which one has been exposed during one's lifetime” (Peretz and Coltheart, 2003, p. 690). When a match for the musical phrase under consideration is found in the musical lexicon, what follows is recognition in the form of a feeling of familiarity and/or possible recall of associated information such as song title or composer, or autobiographical details from one's past life. In this study, we associate the musical lexicon with musical semantic memory. Double dissociations found in studies of deficits in patients with brain damage or congenital disorders suggest that recognition of music involves at least some brain areas that are distinct from those involved in recognition of other types of auditory input (Peretz et al., 1994; Peretz, 1996, 2001; Ayotte et al., 2000).

In an fMRI study, Peretz et al. (2009) found that listening to familiar music, compared to unfamiliar music, correlated with activity in the right superior temporal area. A voxel-based morphometry analysis of patients with semantic dementia also found that the amount of atrophy in this region, especially in the right superior temporal pole, was associated with a reduced ability to recognize famous tunes (Hsieh et al., 2011). When using a familiar melody pitch error detection task to test melody recognition in a combined group of patients with frontotemporal dementia, semantic dementia, and Alzheimer's disease, as well as healthy controls, Johnson et al. (2011) found that recognition correlated with gray matter volume of right-sided brain areas in the inferior frontal gyrus, inferior and superior temporal gyri, and the temporal pole. Interestingly, performance in this task was not correlated with any neuropsychological tests, whereas recall of familiar melody titles did correlate with tests requiring semantic knowledge. This suggests that the title recall may be related to non-musical semantic processing (due to the observed correlation with standard neuropsychological tests of semantic memory), whereas the familiar melody pitch error detection task may be exclusively testing the musical aspects of melody recognition (which were not part of the standard tests).

Groussard et al. (2010), when comparing musical against verbal familiarity in an fMRI study, similarly identified increased activation in the superior temporal gyrus. In the same study, activation within a large network, including the inferior frontal, posterior inferior and middle temporal, medial superior frontal gyri, and the right superior temporal pole, was associated with the level of musical familiarity. The authors suggest that the left superior temporal gyrus may be involved in access to, and the left inferior frontal area in selection from, musical semantic memory, whereas the right superior temporal gyrus activation may reflect retrieval of perception-based musical memory. In a study of familiarity with music and odor stimuli, Plailly et al. (2007) found a largely left-sided activation related to music familiar to the participant (compared to unfamiliar) in several regions of the frontal gyrus, superior temporal sulcus, cingulate, supramarginal, precuneus, mid-occipital, and right angular gyri. Certain regions of activation were common to both olfactory and musical familiarity, but the superior temporal sulcus was among those exclusive to musical familiarity^1^.

None of the aforementioned studies on the neural basis of tune recognition were conducted with older adults. In non-musical domains, neural activation for memories is thought to undergo an age-related shift from medial temporal lobe structures such as the hippocampus and entorhinal cortex to neocortical areas such as the prefrontal cortex (Haist et al., 2001; Douville et al., 2005; Frankland and Bontempi, 2005; Smith and Squire, 2009; Squire and Wixted, 2011). A similar shift is also possible for musical memory. The goal of the present study was to determine if this age-based difference might also occur in musical memory. To this effect, we used fMRI to investigate the neural correlates of melody recognition in younger and older adults. In order to isolate this process as much as possible, we contrasted the brain activity while listening to familiar melodies against listening to unfamiliar melodies, where the unfamiliar melodies had many of the same musical properties as the familiar melodies, yet remained unrecognizable. This paradigm was also used by Peretz et al. (2009). Recognition of familiar melodies across groups was expected to be associated with predominant activation in the superior and middle temporal as well as inferior frontal regions; for the superior temporal area, we expected a stronger activation on the right. Based on evidence of the effects of aging on the neural correlates of semantic memory in non-musical domains, we expected older adults (relative to younger adults) to have increased activity in the prefrontal cortex, specifically the inferior frontal gyrus, and reduced activity in the superior temporal region and in medial temporal lobe structures, i.e., the hippocampus and surrounding areas. A reduced dependence on the medial temporal lobe would provide a plausible neuronal basis for preserved musical memories in conditions where function in this region is compromised, such as starting early in Alzheimer's disease.

## Materials and methods

### Participants

All participants were female, right-handed, and non-musicians, defined for our purposes as those with a maximum of 3 years of music training. Younger (*n* = 20; age range = 18–25 years, mean = 20) and older (*n* = 20; age range = 65–84 years, mean = 71) adults did not differ in years of education [*M* = 14.1 and *M* = 14.7, respectively, *t*_(38)_ = 1.1, *p* = 0.29]. Older participants scored in the normal range on the Mini-Mental State Examination (Folstein et al., 1975; *M* = 29.5, *SD* = 0.87, range = 27–30).

### Stimuli

The familiar and unfamiliar tunes had been tested for familiarity in a pilot study. On a familiarity scale from 0 (“not-at-all familiar”) to 3 (“definitely familiar”), where the level of familiarity indicated the certainty of the participant having heard the tune anywhere during their lives, a cut-off of 1.5 for familiar vs. unfamiliar tunes was used for younger and older participants who met the same criteria as in this study.

**Figure 1:**
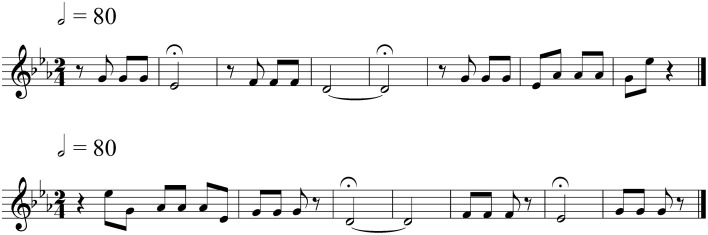
**An original (familiar) melody and its reversed (unfamiliar) version**. The order of the notes in the familiar melody, an excerpt from Beethoven's “Symphony No. 5” **(top)**, is reversed to create an unfamiliar melody **(bottom)**.

Familiar melodies were excerpts from well-known instrumental pieces without associated lyrics in order to avoid stimulating brain activity specifically related to language use. Unfamiliar melodies were reversed versions of familiar melodies, i.e., with reversed note order and preserved tempo, thus largely matching the tonal and temporal qualities of their original counterparts in order to limit the difference between the two versions to familiarity as much as possible (Hébert et al., 1995). Minor corrections were made to the unfamiliar melodies, when required, due to any irregularities in the metrical structure^2^. It was important to maximally control for perceptual differences between the familiar and unfamiliar melodies while ensuring that the unfamiliar melodies were unrecognizable. Figure 1 shows a sample melody and its reversed version. A complete list of familiar melodies used in this study is given in the Appendix. The selected familiar and unfamiliar melodies were similar in style to those used by Peretz et al. (2009).

Four types of stimuli were used during the functional imaging: familiar melodies, unfamiliar melodies, signal-correlated noise (SCN), and silent trials. Each stimulus was 8.5 s in length, including a 500 ms fade-out at the end of the melodies. The melodies were edited using Sibelius^3^ v6 music notation software, and exported as wave files using Kontakt Player 2 by Native Instruments^4^ running the Steinway Grand Piano sample bank by Garritan Personal Orchestra^5^ v3. The sound files were encoded with 16 bits per sample at a rate of 44.1 kHz using Audition v3 by Adobe^6^. The SCN stimuli were created with Praat software (http://www.praat.org) from randomly selected unfamiliar melodies. SCN preserves the amplitude envelope and spectral profile of a waveform (Davis and Johnsrude, 2003), but does not contain spectral details of the sound. SCN was used as a reference stimulus for the melodies since it retains some of the temporal patterns and pitch from the original melody while remaining unrecognizable.

### Procedure

Each participant was tested over two sessions on separate days. The main goal of the first session was to familiarize participants with the experimental setup by using a sham magnetic resonance (MR) system^7^ to simulate a block of the functional imaging protocol. Participants also completed demographic, health, and music-related questionnaires and cognitive tests (older adults only) during the first session. All scanning occurred during the second session. A total of 120 stimuli (39 familiar tunes, 39 unfamiliar tunes, 21 signal-correlated noise, and 21 silent trials) were presented in a pseudorandomized order, divided into four blocks containing 30 stimuli each, with roughly the same numbers of each stimulus type in each block. The participants were asked to simply listen to the stimuli, without providing any active responses, in order to avoid activation related to decision-making processes or motor activity related to button presses. After the scanning session, the participants listened to the same melodies as in the scanner to rate their level of familiarity for each tune. Participants were instructed to base the rating on general lifetime familiarity, and not on whether the tune had been heard in the scanner. The rating scale ranged from 0 (“not-at-all familiar”) to 3 (“definitely familiar”)^8^.

A Siemens 3 Tesla MAGNETOM Trio whole body MRI scanner with a 12-channel head matrix coil was used for the scanning. The auditory stimuli were delivered through a NordicNeuroLab Audio System with headphones that also provided a noise attenuation of approximately 30 dB^9^. Detailed structural images were obtained at the start of scanning using a T1-weighted magnetization-prepared rapid gradient echo (MP-RAGE) single-shot sequence to acquire 176 sagittal slices with a field of view (FOV) = 256 × 256 mm, in-plane resolution = 1.0 mm × 1.0 mm, slice thickness = 1.0 mm, flip angle (FA) = 9°, repetition time (TR) = 1760 ms, echo time (TE) = 2.2 ms. This scan took 7.5 min. After the structural scan, a sample tune was played to allow adjustment of the sound intensity to a level comfortable for the participant listening through the headphones. The sparse-sampling functional imaging protocol was then started. A single brain volume was acquired immediately following each 8.5-s stimulus. Scans occurred every 10.5 s using a T2^*^-weighted GE-EPI interleaved sequence to acquire 32 axial slices with FOV = 211 × 211 mm, in-plane resolution = 3.3 × 3.3 mm, slice thickness = 3.3 mm with a 25% gap, FA = 78°, acquisition time = 2000 ms, TE = 30 ms, TR = 10.5 s. In this way, sounds were presented in the silent intervals between successive scans, and scans were acquired at a point in time at which the hemodynamic response to the stimulus would be measurable.

During the functional imaging, 120 volumes were obtained over the course of 4 blocks that lasted 5.6 min each.

### Imaging data analysis

SPM^10^ software was used to analyze imaging data. For each participant, processing was performed using the following steps, in the given order: (1) DICOM to NIfTI conversion; (2) spatial preprocessing: realignment (motion correction), co-registration of the functional time-series with the T1-weighted structural image, segmentation and normalization of the T1-weighted image to MNI152 space, application of the T1-derived deformation parameters from the normalization step to the fMRI time series; and smoothing using a Gaussian kernel with a FWHM of 8 mm (3) model specification and estimation; and (4) contrast specification and generation of statistical parametric maps. The familiar-SCN and unfamiliar-SCN contrasts were computed for each participant. These contrasts formed the basis of a full-factorial model in SPM using melody familiarity as a within-subject factor with two levels (familiar and unfamiliar) and age as a between-group factor with two levels (younger and older adults). The results pertinent to our study were activation related to the main effect of tune recognition and any age group differences in this activation resulting from a familiarity-age interaction. Whole brain and region-of-interest (ROI) analyses were performed for the resulting contrasts. We used the SPM toolkit MarsBaR (Brett et al., 2002) with automated anatomical labeling (Tzourio-Mazoyer et al., 2002) to examine activation in specific anatomical ROIs. Due to our hypothesis, we tested frontal regions for greater recognition-related activity in older adults compared to younger adults, and conversely, we tested hippocampal and superior temporal regions for greater activity in younger relative to older adults. In order to focus on specific areas, we selected the pre-defined anatomical ROIs based on regions showing significant overall recognition-related activation across all participants. Additionally, a regression analysis examined the effect of age on tune recognition activity separately in each of the younger and older groups. In all cases, a family-wise error-corrected *p*-threshold (*p*FWEc) of 0.05 (Worsley et al., 1996) was applied to all statistical probability maps in order to determine significant areas of activation in SPM.

The study was cleared for ethics compliance by the Queen's University Health Sciences and Affiliated Teaching Hospitals Research Ethics Board. All participants gave written informed consent in accordance with this Ethics Board's guidelines and received $30 over two sessions.

## Results

**Figure 2:**
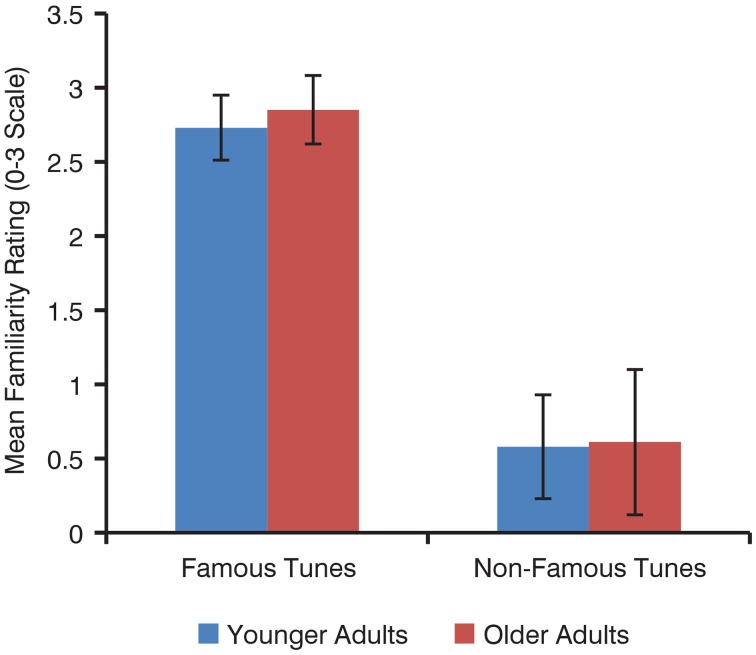
**Mean familiarity ratings of familiar and unfamiliar tunes by younger and older adults**. Error bars represent standard deviation.

There was no effect of age on familiarity ratings of familiar tunes [younger *M* = 2.73, *SD* = 0.22; older *M* = 2.85, *SD* = 0.23; *t*_(38)_ = 1.6, *p* = 0.12] or of unfamiliar tunes [younger *M* = 0.58, *SD* = 0.35; older *M* = 0.61, *SD* = 0.49; *t*_(34)_ = 0.17, *p* = 0.87; Figure 2]. Overall, familiar tunes were rated as more familiar than unfamiliar tunes by all participants [familiar *M* = 2.79, *SD* = 0.19; unfamiliar *M* = 0.59, *SD* = 0.32; *t*_(62)_ = 36.1, *p* < 0.001].

Recognition of a tune was operationalized as a familiarity rating of 2 or 3 for a familiar tune and 0 or 1 for a unfamiliar tune (on a familiarity scale from 0 to 3). Younger and older adults did not differ in percentage of tunes thus recognized [younger *M* = 88.4, *SD* = 7.9; older *M* = 89.8, *SD* = 9.2, *t*_(38)_ = 0.5, *p* = 0.62].

Activation associated with processing of sound was determined in order to verify the accuracy of the design matrix (assignment of conditions to scans) in this study. In the sound-silence contrast, sound comprised all conditions that contained a melody or signal-correlated noise. The individual contrasts were used in a second-level random effects analysis to determine group activation and differences in activation between groups. Whole-brain analysis showed the expected large clusters of activation in the right and left temporal gyri, with respective peaks at MNI x, y, z (in mm) = 49, −24, 10 (*t* = 18.9, cluster size = 1033 voxels) and MNI *x, y, z* (in mm) = −44, −20, 7 (*t* = 17.9, cluster size = 1008 voxels). Younger and older adults did not differ in sound-related activation using whole-brain analysis with a family-wise error corrected *p*-threshold of 0.05.

**Figure 3:**
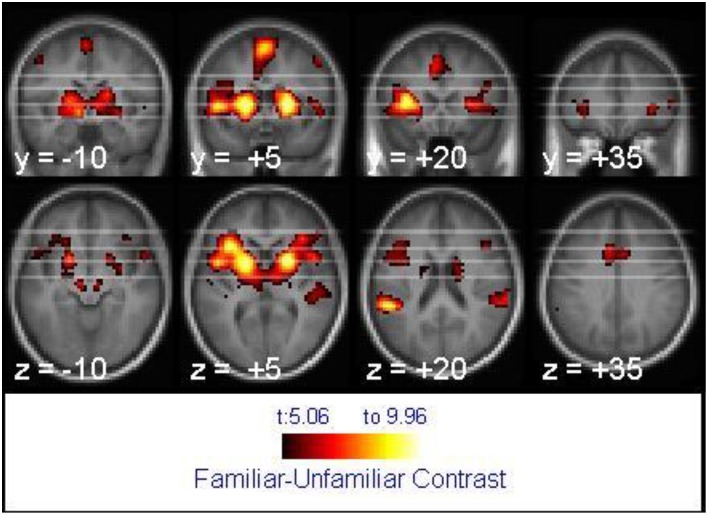
**Activation associated with melody familiarity for all participants**. *p*FWEc < 0.05; *N* = 40. The activation is displayed on the average of all participants' spatially normalized structural images. The right side of each image represents the right side of the brain. Coordinates are in MNI space.

For tune recognition in the whole brain analysis, the 2 × 2 mixed ANOVA showed no main effect of age, but the main effect of melody familiarity yielded several cortical regions of activation. The largest recognition-related activation cluster (2304 voxels) appeared in the bilateral inferior frontal, left middle orbitofrontal, and right superior temporal gyri (Figure 3). Other clusters of activation were found within the left supramarginal, bilateral superior frontal, left cingulate, and bilateral precentral gyri (Table 1).

**Table 1:** **Activation peaks by cluster for melody recognition**.

**Activation Cluster and Peak**	**Cluster Size (voxels)**	***p* FWE-Corrected**	***t*_(39)_**	**MNI Coordinates (mm)**		
				***x***	***y***	***z***
IFG, OFG, STG, thalamus, putamen, brainstem	2304					
L IFG		0.000	9.34	−47	6	4
		0.000	9.16	−41	22	4
		0.000	6.90	−54	9	14
		0.000	6.17	−41	6	24
R IFG		0.000	7.32	39	29	1
		0.000	6.84	49	9	1
		0.001	5.93	45	19	24
L middle OFG		0.000	6.17	−24	26	−9
R STG		0.001	5.99	55	13	−13
		0.010	5.30	55	−7	−6
R thalamus		0.000	7.31	9	−7	4
L putamen		0.000	10.55	−21	6	4
		0.000	7.97	−21	9	−13
		0.000	6.55	−31	−14	−3
R putamen		0.000	9.98	22	9	4
		0.001	5.99	29	−17	−3
brainstem		0.000	7.27	−11	−17	−9
		0.000	6.14	−4	−24	1
		0.001	5.98	9	−24	−3
L Supramarginal gyrus	186	0.000	10.22	−50	−40	24
SFG, Cingulate	511					
L SFG		0.000	9.12	−1	−1	63
		0.000	7.76	−4	13	53
R SFG		0.000	6.42	6	13	40
L Cingulate gyrus		0.000	6.96	−8	13	37
R STG	152	0.000	7.29	65	−34	14
L Precentral gyrus	69	0.000	6.70	−50	−7	47
		0.000	6.45	−44	−4	57
R Precentral gyrus	54	0.000	6.66	52	3	50

*pFWEc < 0.05; N = 40*.

*These results are from whole-brain analysis in SPM. Peaks are grouped by cluster. Only clusters containing more than 50 voxels are listed. All peaks separated by a minimum distance of 10 mm are included. Labels for the peak coordinates are from the LPBA40 atlas. FWE, family-wise error; L, left; R, right; IFG, inferior frontal gyrus; OFG, orbitofrontal gyrus; STG, superior temporal gyrus; SFG, superior frontal gyrus*.

**Figure 4:**
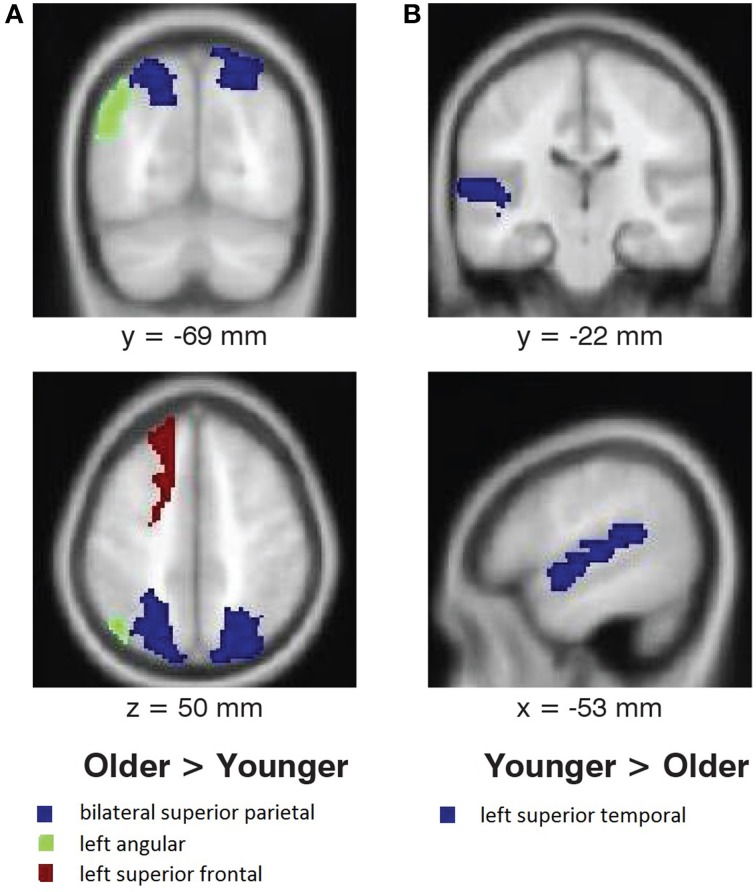
**Age differences in activation associated with melody familiarity**. *p*FWEc < 0.05; ROI Analysis using MarsBaR. **(A)** Regions where activation was greater for older adults than younger adults, and **(B)** regions where activation was greater for younger adults than older adults. For the coronal and axial slices, the right side of the image represents the right side of the brain. Coordinates are in MNI space.

No interaction between melody familiarity and age was found for whole brain analysis, i.e., there were no differences between younger and older adults in recognition-related activation. The regression analysis for the main effect of age on tune recognition also yielded no regions of significant activation in either the younger or the older groups. However, ROI analysis in MarsBaR (Brett et al., 2002) on cortical areas with overall recognition-related activation (Table 1), when tested bilaterally, did show some group differences (Figure 4). Younger adults had greater recognition-related activation than older adults in the left superior temporal gyrus [*t*_(38)_ = 2.27, *p* = 0.013], whereas older adults showed greater activation than younger adults in the left superior frontal gyrus [*t*_(38)_ = 1.81, *p* = 0.037]. In accordance with our hypothesis of greater recognition-related activation in the medial temporal lobe in younger adults, we tested the hippocampal region. Although the right hippocampus did have greater activation in younger adults, the results were not significant [*t*_(38)_ = 1.49, *p* = 0.070]. Lastly, an exploratory analysis on the parietal region showed greater recognition-related activity in older adults in the left angular gyrus [*t*_(38)_ = 3.35*, p* < 0.001] and bilateral superior parietal gyrus [*t*_(38)_ = 1.85, *p* = 0.034].

## Discussion

We found robust activation for familiar melodies (compared to unfamiliar melodies) in several frontal lobe areas, along with the superior temporal and supramarginal gyri, when testing the full set of 40 participants from the two age groups conjointly. The specific frontal areas of activity were in the bilateral inferior frontal, left middle orbitofrontal, and bilateral superior frontal gyri. Subcortical regions with recognition-based activation were in the thalamus, putamen, and brainstem. ROI analysis showed greater activation in the left superior temporal gyrus for younger adults and in superior frontal, angular, and superior parietal regions for older adults.

Activation associated with familiar melodies must be understood in the context of the full experience of the participant during the imaging. Recognition of a familiar melody can sometimes be accompanied by recall of a specific incident when the melody may have been heard or of other memories associated with the melody. Since unfamiliar melodies are less likely to evoke comparable memories, the recognition-related activation could include episodic memory or perhaps details about the melody itself, such as the composer. In addition, familiar melodies may be associated with specific emotions. Thus, activation in brain regions that are known to be involved in these functions may be attributed to the corresponding aspects of the participant experience instead of strict access to musical memory itself.

The regions of activation associated with tune recognition in this study have also been identified in various previous studies of musical semantic memory. The inferior frontal and superior temporal areas, which were part of the largest cluster (2304 voxels) of activation, have been frequently reported. The inferior temporal region (Halpern and Zatorre, 1999; Platel et al., 2003; Satoh et al., 2006; Plailly et al., 2007; Groussard et al., 2010) may participate in retrieval from semantic memory in both musical (Groussard et al., 2010) and non-musical (Binder et al., 2009) domains. According to Binder and Desai (2011), the left inferior frontal gyrus activation occurs during selection from semantic memory from among several alternatives.

The role of the superior temporal region has been established in numerous studies of musical semantic memory (Halpern and Zatorre, 1999; Platel, 2005; Satoh et al., 2006; Plailly et al., 2007; Groussard et al., 2009; Janata, 2009; Peretz et al., 2009; Hsieh et al., 2011; Pereira et al., 2011). This region may be unique to the musical domain. When examining non-musical semantic memory in a meta-analysis of 120 fMRI studies, Binder et al. (2009) found little evidence of superior temporal involvement. This region is also involved in processing of melodic sounds in general (Patterson et al., 2002), including pitch contours (Lee et al., 2011). Thus, some of the superior temporal activation may reflect the use of the melodic information to access the musical lexicon. Our results partially replicate those of Peretz et al. (2009) who identified a possible basis of the musical lexicon in the right temporal sulcus. The location of their peak activation in this region is just inferior to the one in our study. Peak coordinates in the study by Peretz et al. (2009) were at MNI *x, y, z* = 48, −24, −10; the corresponding peak in this study was at MNI *x, y, z* = 52, −20, 1; Euclidean distance between these two peaks being 12.4 mm.

Other frontal regions of recognition-related activation were in the middle orbitofrontal, superior frontal, and precentral gyri. The orbitofrontal cortex was also identified by Groussard et al. (2010) and Hsieh et al. (2011) for musical memory. This region is a multimodal association area where activity is related to subjective pleasantness (Kringelbach and Radcliffe, 2005) and may therefore, reflect enjoyment of familiar tunes. The superior frontal gyrus, which has been previously associated with musical semantic memory (Platel, 2005; Groussard et al., 2010), may underlie top-down access to knowledge for intentional retrieval (Schott et al., 2005; Binder and Desai, 2011). The precentral gyrus activation may be due to subvocalization, e.g., humming along to familiar music.

Supramarginal activation was found in a study comparing memory for music and odors (Plailly et al., 2007). This region may be involved in maintaining memory for musical pitch (Schaal et al., 2015), and may correspond to recalling the notes of a familiar melody. Subcortical activation in the putamen and brainstem may be associated with motor synchronization to rhythm that is more active when engaging with familiar music (Pereira et al., 2011). It may reflect top-down feedback in the auditory pathway from the cortex to lower nuclei such that familiarity leads to a greater response due to anticipation for the familiar tune. The thalamus may play a similar role for familiar melodies since thalamic activation, having both afferent sensory and efferent motor connections, is linked to task performance, even for very simple tasks, possibly through ongoing changes in motivation and arousal (Schiff et al., 2013).

The main goal of this study was to investigate age differences in the neural basis of melody recognition. Region-of-interest analysis showed that younger adults had greater activation in the left superior temporal gyrus whereas older adults exhibited increased activity in the left superior frontal gyrus and bilateral parietal areas. Because these findings represent new data in the field of aging effects on the neural basis of musical memory, comparisons can only be made to non-musical memory studies where some of the regions involved are distinct from those involved in musical memory. According to the standard consolidation theory, memories become consolidated through stronger cortico-cortico connections over time (Frankland and Bontempi, 2005). The increased activity in the frontal and parietal areas in older adults may result from such consolidation. In a study with famous names, older adults had more activation in the superior frontal and middle temporal regions for “enduring” names (of people who had been for some time and continued to be famous) than non-famous names. Grady (2012) interprets the additional age-related recruitment of frontal areas in general as a compensatory mechanism.

In view of the standard consolidation model of memory, which proposes that memories become less dependent on the medial temporal lobe over time (Frankland and Bontempi, 2005), we expected to find a greater hippocampal region activation in younger adults than in older adults. Younger adults had presumably acquired knowledge of the largely classical and popular tunes more recently than the older adults. A greater activation (marginally significant at *p* = 0.07) in the right hippocampus of younger adults as opposed to older adults promotes further hypotheses. For example, if the melodies for the adults were from a remote period and unlikely to have been heard more recently, there may be a sharper division between the recent and remote natures of the memories for younger and older adults, respectively. In that case, however, it would be important to ensure a close match, as was done here, in the acoustical and musical properties of the two sets of melodies.

In this study, we have identified the neural basis of melody recognition in a network comprising cortical regions within the superior temporal, inferior and superior frontal, middle orbitofrontal, supramarginal, and precentral gyri. We have shown that older adults engage additional resources in superior frontal and parietal areas whereas younger adults preferentially employ the superior temporal region. Our study makes a reliable contribution to the knowledge of the neural basis of melody recognition based on a large sample size of 40 participants (20 older, and 20 younger, adults). This is also the first study (to the best of our knowledge) to compare brain activity associated with musical semantic memory in younger and older adults.

### Conflict of interest statement

The authors declare that the research was conducted in the absence of any commercial or financial relationships that could be construed as a potential conflict of interest.

## Footnotes

^1^These studies of familiar tunes are testing remote (lifetime) memory for music, whereas in memory literature, the terms “familiarity,” “recollection,” and “recognition” most frequently refer to studies where participants study a list of items and are then tested on these items (Yonelinas, 2002), usually in the same session (i.e., a few minutes later). Familiarity in this study-test paradigm refers to knowing whether an item was on the study list. The participant may have lifetime familiarity with ALL the items (e.g., famous faces, or random words), but still not be “familiar” with the item in the sense of having seen it in the study list. This type of memory is likely different than “lifetime familiarity”; the latter is the focus most music recognition studies, including this one.

^2^All melodies were examined and verified as musically correct by Bernard Bouchard, a composer from the BRAMS Laboratory at the University of Montreal (http://www.brams.org/en/membres/bernard-bouchard/). He also created the stimuli for a similar study on familiar music with younger adults (Peretz et al., 2009).

^3^http://www.sibelius.com/.

^4^http://www.native-instruments.com/.

^5^http://www.garritan.com/.

^6^http://www.adobe.com/.

^7^The sham MR system has a bore with the same dimensions as the real MRI scanner, as well as a similar table that slides into this opening. The scanning sounds were simulated by playback of the appropriate scan sequence from a CD supplied by the scanner manufacturer. The sound pressure level of the scanning sound simulations was approximately 90 dB, approaching the volume of the MRI scanner.

^8^The endpoints of this 4-point scale implied that the participant was completely certain, whereas the two points in the middle indicated some uncertainty, about whether they had encountered the tune before. An even number of options allowed us to learn which side of the familiar or unfamiliar question best fit their feelings toward a tune.

^9^http://www.nordicneurolab.com/.

^10^SPM8 from Functional Imaging Laboratory at Wellcome Trust Centre for Neuroimaging, http://www.fil.ion.ucl.ac.uk/spm/, running on MATLAB 2010a from MathWorks, http://www.mathworks.com/products/matlab/.

## Appendix

### Familiar tunes

**Table:**

**Stimuli #**	**Composer**	**Title**	**Starting bar #**
1	Peter Ilyich Tchaikovsky	1812 Overture, Op. 49	78
2	Ludwig van Beethoven	Symphony No. 5 in C Minor, Op. 67, Movement 1	1
3	Luigi Boccherini	Minuet from String Quintet in E Major, Op.11, No.5 (G275)	1
4	Georges Bizet	Carmen Suite No.1, Movement 6 “Les Toréadors”	55
5	Vangelis	Chariots of Fire	11
6	Werner Thomas	The Chicken Dance	1
7	Johann Strauss Jr.	An der schönen blauen Donau, Op. 314 (“The Blue Danube Waltz”)	45
8	Wolfgang Amadeus Mozart	Serenade No. 13 for strings in G Major, K. 525 “Eine Kleine Nachtmusik”	1
9	Scott Joplin	The Entertainer	4
10	Robert Schumann	Fröhlicher Landmann, von der Arbeit zurückkehrend (The Happy Peasant—Le gai laboureur)	1 (pickup beat)
11	Frédéric Chopin	Piano Sonata No. 2 in B flat minor, Op. 35, Movement 3”Marche funèbre: Lento”	3
12	Ludwig van Beethoven	Bagatelle No. 25 in A minor (WoO 59 and Bia 515) “Für Elise”	1 (pickup beat)
13	Edvard Grieg	Peer Gynt Suite No.1, Op.46, Movement 1 “Morning Mood”	1
14	Edvard Grieg	Peer Gynt Suite No.1, Op. 46, Movement 4 “In the Hall of the Mountain King”	2
15	Johannes Brahms	Hungarian Dance No.5	1
16	Jacques Offenbach	Orphée aux enfers (Orpheus in the Underworld), The “Infernal Galop” from Act II, Scene 2	219
17	Glenn Miller	In the Mood	1
18	Irish Traditional Music	The Irish Washerwoman/ The Washwoman	1 (pickup beat)
19	Johann Sebastian Bach	Cantata BWV 147, Herz und Mund und Tat und Leben, Movement 10 “Wohl Mir, Dass Ich Jesum Habe” (Jesu, Joy of Man's Desiring)	1
20	Ludwig van Beethoven	Piano Sonata No.14 in C Sharp Minor, “Quasi Una Fantasia” Op. 27, No.2 (Moonlight Sonata)	42
21	Peter Ilyich Tchaikovsky	Marche (March) from The Nutcracker	1
22	Henry Mancini	Theme Song from The Pink Panther	11
23	Edward Elgar	Pomp and Circumstance. Military Marches. Op. 39, No. 1 in D Major “Trio”	78
24	Mexican Folk Dance	La Raspa	1
25	Peter Ilyich Tchaikovsky	Les Mirlitons (Dance of the Reed-Pipes) from The Nutcracker	3
26	Wolfgang Amadeus Mozart	Piano Sonata No.11 in A Major, K. 331 (300), Movement 3 “Alla Turca”	1
27	Aram Khachaturian	Sabre Dance from Gayane	3
28	Paul Dukas	The Sorcerer's Apprentice (L'Apprenti Sorcier)	72
29	John Williams	Star Wars Main Theme	1 (pickup beat)
30	Peter Ilyich Tchaikovsky	Danse de la Fée-Dragée (Dance of the Sugar Plum Fairy) from The Nutcracker	5
31	Peter Ilyich Tchaikovsky	Swan Lake Op. 20, Act II, No. 10 Scène: Moderato	2
32	Wolfgang Amadeus Mozart	Symphony No. 40 in G minor, KV.550, Movement 1 “Molto Allegro”	1
33	Julius Fučík	Einzug der Gladiatoren, Op. 68 (“Entry of the Gladiators” or “Thunder and Blazes”)	13
34	Georges Bizet	Carmen Suite No.1, Movement 6 “Les Toréadors”	1
35	Peter Ilyich Tchaikovsky	Trepak (Russian Dance) from The Nutcracker	1
36	Richard Wagner	Die Walküre, WWV 86B, Act III	14
37	Antonio Vivaldi	Concerto No.1. La Primavera, Spring, Frühling. Op.8, No.1, RV 269, Allegro	1 (pickup beat)
38	Peter Ilyich Tchaikovsky	Valse des Fleurs (Waltz of the Flowers) from The Nutcracker	38
39	Gioachino Rossini	William Tell Overture, Finale, March of the Swiss Soldiers	243
